# The public health importance of scabies in community domiciliary care settings: an exploratory cross-sectional survey of Health Protection Teams in England

**DOI:** 10.1017/S0950268819001274

**Published:** 2019-07-10

**Authors:** Emily Phipps, Maaike. E. Pietzsch, Jackie A. Cassell, Clare Humphreys

**Affiliations:** 1 Oxford School of Public Health, England; 2 NIHR Health Protection Research Unit in Emerging and Zoonotic Infections, UK; 3 Public Health England South East, England; 4 Brighton and Sussex Medical School, England

**Keywords:** Communicable disease control, domiciliary care, health protection, public health, scabies

## Abstract

Scabies is a contagious skin infection commonly occurring in institutions such as care homes. However, a large proportion of vulnerable people in England receive domiciliary care in the community and their experience of scabies has not been described. We undertook a pragmatic cross-sectional survey of Health Protection Teams (HPTs) in England to determine the burden of scabies related to domiciliary care. Fifteen cases or outbreaks were notified to HPTs between January 2013 and December 2017. Although a relatively uncommon event for individual HPTs, they were complex to manage and required the co-ordination of multiple stakeholders. Diagnosis was often delayed and required several clinical consultations. A lack of guidance led to difficulties establishing stakeholder roles and responsibilities and sources of funding for treatment. The stigmatisation of scabies sometimes affected the quality of care provided to patients, such as use of excessive personal protective equipment. Our study demonstrates that scabies is an issue of public health importance for domiciliary care service providers and users, and research is required to better understand the impacts of the disease and to develop evidence-based guidance. More generally, there is a need for simpler treatment regimens and methods of diagnosing scabies.

## Introduction

Scabies is a highly contagious skin condition caused by burrowing *Sarcoptes scabiei* mites [1]. It causes intense and prolonged itching, and excoriations can lead to secondary bacterial infections [2]. It is primarily transmitted through touch, and so institutions in which extended skin to skin contact is common place (such as hospitals and care homes) carry a considerable burden of disease in high-income countries [3, 4].

Outbreaks of scabies in care home settings have been widely documented and are often difficult to recognise and manage, especially in residents with communication difficulties or dementia [4, 5, 6]. Close and frequent physical contact between carers and residents is frequent in order to meet personal and health care needs, while the long asymptomatic incubation period of 2–6 weeks means that many staff and residents can become infected through this route before the outbreak is recognised [5]. Control of an outbreak requires co-ordinated treatment with topical creams of all cases and contacts, which can be logistically challenging, resource intensive and particularly distressing for residents with dementia who might not understand the reason for treatment [4]. Systemic treatment with a single dose of ivermectin has been demonstrated to effectively treat scabies without the need for topical creams [7], however it is unlicensed for this use in the UK and is only currently recommended in combined use with creams for severe cases that do not respond to topical treatment.

While there is strong evidence of scabies being an important public health concern for vulnerable residents in care homes [4, 7, 8, 9], most people receiving social or nursing support in England do not live in these facilities. In 2015, 273 124 people in the United Kingdom received care in their own homes (‘domiciliary care’) from nurses or care assistants funded either privately or by a local authority [10]. The exposure routes and risk of transmission through physical contact will be the same as in residential facilities, but the disease burden and best public health practice in this context have not yet been explored.

To date, there are very few case reports in high-income countries on scabies outbreaks associated with domiciliary care in the literature [11, 12]. In 2000, Andersen *et al*. reported a case series of an outbreak of scabies in three Norwegian nursing homes which also affected six domiciliary care service users looked after by the same staff [12], while in 2012, Ladbury *et al*. identified home care agencies as being associated with transmission between four cases of scabies in a cluster of 26 in the Netherlands [11]. However, there have been no reports of scabies associated with domiciliary care in England published in the peer-reviewed literature, and there is no national public health guidance on the management of scabies outbreaks available.

Recognising this evidence gap, we undertook a scoping study which aimed to explore the potential burden of scabies cases and outbreaks associated with domiciliary care in England to better understand how these outbreaks are currently managed, in order to support further research and the development of appropriate public health guidance.

## Methods

In the English public health system, Health Protection Teams (HPTs) are locally placed teams, providing operational support to the National Health Service (NHS), local authorities and other agencies. They are responsible for surveillance and investigation of health protection incidents and lead on Public Health England's (PHE) response to all health-related incidents, providing specialist advice and support on infectious diseases, chemical and radiation incidents and emergencies. Within the four PHE regions (North of England, South of England, Midlands & East of England and London), there are 15 HPTs who would be involved in the management of scabies outbreaks. Given how little is known on the subject, we employed a pragmatic approach of surveying HPTs as these were accessible to us through professional networks. We aimed to ascertain how many scabies outbreaks associated with domiciliary care were notified to HPTs for support in management, in order to establish whether this is an issue warranting public health concern and to identify areas for future research.

An online survey was developed using the tool SelectSurvey (ClassApps Inc © Copyright 1998–2018). Survey items drew on notes on scabies cases and outbreaks handled by PHE Thames Valley HPT which covers Berkshire, Buckinghamshire and Oxfordshire. The records for cases and outbreaks documented on the health protection management system HPZone (inFact Shipley Ltd 2012) and existing literature on scabies outbreaks in care homes [4, 5] were reviewed by an author (EP), and the key themes were adapted to open and closed questions for the survey (see online Supplementary material for the survey tool).

Participants were asked to conduct a search of the health protection management system HPZone (inFact Shipley Ltd 2012) to identify all cases or outbreaks of scabies notified between 1 January 2013 and 31 December 2017 where a case was either a domiciliary care worker, or received care from domiciliary care services.

The survey was piloted with three HPTs in the South East covering Kent, Surrey and Sussex, Hampshire and the Isle of Wight. Responses from pilot participants were reviewed to ensure clarity and relevance of themes.

Following the pilot, all HPTs in England were approached to take part in the study through the UK Public Health Network for Zoonoses and PHE centre health protection meetings. Whilst scabies is not zoonotic, the former group was considered to have appropriate membership with experience or familiarity of vector-borne infections and links to all HPTs across England. The survey instructions and website link (see online Supplementary material for a copy of the instructions) were circulated through the group and potential participants were asked to contact the study team for further information. The name of the person completing the survey, their role and HPT were collected in order to facilitate follow-up if needed and to avoid duplication of responses. The HPZone reference number for all identified cases and outbreaks were also collected for this reason. This unique reference number was stored on a secure PHE computer separate to any linked identifiable information.

Descriptive statistics were generated to compile responses to the quantitative, close-ended and multiple-choice questions. Free-text responses to the open-ended questions were reviewed to identify codes and themes for discussion.

As this survey examined how standard public health care was delivered, and findings were not intended to be generalisable or transferrable to other patient groups or settings, it was determined to be a service evaluation study by the PHE Research and Ethics Governance Group and research ethics approval was not required.

## Results

The response rate to the survey was 67% (*n* = 10/15). These participating HPTs were located in the North West, North East, South West, South East and the Midlands regions of England. Responses were received from HPTs across England describing a total of 15 incidents or outbreaks related to scabies and domiciliary care. Data completeness was generally good, with data on between 13 and 15 incidents captured for the majority of questions in the survey.

The average number of individual cases or outbreaks of scabies handled by each HPT between 1 January 2013 and 31 December 2017 was 1.6 (median 1.0, range 0–6). All reported incidents and outbreaks included more than one case of scabies, apart from one which concerned a single case of scabies in a care worker with no affected service users identified following clinical assessment.

### Delay in diagnosis and notification

The median length of time between symptom onset in the first case and notification to the HPT was 6 weeks (mean 10 days, range 0.4–52 days). HPTs were involved in cases/outbreaks for an average of 14 days (median 4, range 1–90). Internal record keeping by some care agencies was limited, making it slow and difficult to identify potentially exposed carers and service users. The average number of care workers affected, either because they were a confirmed case of scabies or had been exposed to one, was 12 (range 1–100) and service users five (range 1–60). Many respondents commented about the significant length of time between symptom onset and diagnosis, with service users having several clinical consultations before a diagnosis was made.

### Multiagency working

An outbreak control team (a multi-disciplinary team brought together for expert input in to outbreak management) was convened for three outbreaks. Many participants commented that involvement in the management of these scabies cases and outbreaks presented an excellent opportunity for multi-agency working and relationship building with new stakeholders from the private sector. When community infection control nurses (providing organisational- and individual-level infection control support and guidance) were involved, their knowledge of the local community health economy and provision of logistical support were seen as important factors in successful cases and outbreak management. When GP practices and domiciliary care agencies were engaged with the process and happy to co-ordinate treatment (and source funding for persons not eligible for free prescriptions), this made outbreak response much easier and more straightforward.

### Funding

There was often a lack of clarity as to who would fund treatments, particularly for care workers, who sometimes had to fund their own prescriptions. Funding for treatment of cases either came from the care agency itself or was funded personally by staff or service users through their usual prescription route (including eligibility for free prescriptions). Unfortunately, the high proportion of missing data means that we are unable to generate summary statistics on this issue.

### Appropriate risk assessment

There was documented evidence of discussions between HPTs and the notifying clinician regarding confidence in the diagnosis reported by 71% of participants, 84% regarding treatment advice and 53% regarding appropriate exclusion. Some also reported that GPs requested support from specialist dermatology services as they were not confident in diagnosing scabies clinically and did not know how to take skin scrapings for microscopy.

### Coordinating treatment

Domiciliary care agencies often have many staff, caring for many service users over a wide geography. This makes organising and delivering co-ordinated treatment, which is essential to break the transmission cycle, very challenging. Treatment for domiciliary care staff or service users was reported as being undertaken in 36% of records. Topical treatment was used in all cases. Co-ordinated treatment was advised in 71% of records, but only documented as being executed in 36%.

### Stigma

Stigmatising management of scabies was an often documented issue reported by respondents. Some reported that domiciliary care agencies refused to provide further care for service users until the infection was resolved, resulting in a significant gap in care provision for these vulnerable adults. There were also reports of the use of excessive personal protective equipment relative to the infection risk to protect against perceived risk of infection, reinforcing stigma regarding scabies. There were also reports of care agencies requesting treatment for all of their staff – in one case over 100 people – due to fears over potential transmission risk.

### Lack of awareness (lack of guidance)

Where guidelines were reported to have been used, these were local or limited national guidelines related to scabies outbreaks in care homes. The reference for guidelines used was only known for five incidents and were local internal HPT guidelines (*n* = 2), NHS Choices page on scabies (*n* = 1), locally available council care home guidelines (*n* = 1) and the PHE care homes infection control guidance (*n* = 1). Virtually all respondents commented that a lack of specific guidance on the subject needed addressing.

## Discussion

In this paper, we have described the characteristics of 15 incidents of scabies related to domiciliary care over 4 years in England that were notified to HPTs. Although a relatively uncommon event for individual HPTs, they were complex to manage and involved the co-ordination of multiple stakeholders, demonstrating that scabies infection associated with domiciliary care is an important public health issue that warrants further study.

Although similar in the route of transmission between carer and service user, the context of domiciliary care is different from nursing or residential homes where current reporting and guidance is mainly focussed. Limited reports from European studies, along with this survey, suggests the outbreaks related to domiciliary care are potentially more difficult to detect and contain. Domiciliary staff go out to visit many clients and may also work in residential care homes. Other agencies providing other services, for example, district nurses, also go in to these homes, and service users may live with other people who provide support requiring physical contact. These factors will make it very difficult to identify everyone at risk and co-ordinate treatment in order to break the cycle of transmission. HPTs provide a substantial amount of input to support the management of what should be a straightforward skin infection, and this is largely down to a respondent reported general lack of knowledge and inadequate guidance on this challenging diagnosis. Global diagnostic criteria for scabies have recently been published, however these will likely not be applicable to older populations, given that presentation differs from traditional classical descriptions in this population group [5, 14]. There is a need for more general guidance around scabies in its various presentations, and its management to help reduce the associated stigma.

As demonstrated in previous literature [4, 5, 11, 12], our findings indicate that there is a considerable delay in diagnosis and notification of scabies in older people with care needs, due to difficulties in recognising and confirming the infection. Such delays are likely to compound potential outbreaks and could allow further spread in the household and into the community – our survey demonstrates that a significant number of carers and service users are exposed and/or infected with scabies before the outbreak is notified to health protection. Furthermore, the lack of clear guidelines and agreement from stakeholders make the process of outbreak management complex and unclear. There is a distinct lack of knowledge and understanding regarding roles, responsibilities and funding arrangements to manage outbreaks once they are suspected or identified. This risks slowing down treatment and continuing the cycle of infection, or puts the onus on care agency employees to fund their own prescriptions for an infection acquired through their employment. Level of access to occupational health services for domiciliary care workers were not explored in this study, but could be a significant barrier to accessing treatment for staff.

There was a variation between HPTs in terms of advice given and action taken to manage cases and outbreaks because there is variation between local guidelines and a lack of clear national guidance [13]. The provision of national public health guidelines on scabies outbreak management is needed to standardise the approach taken by local HPTs, as the current practice of adapting the brief section on scabies in the PHE Handbook for Infection Control in Care Homes [14] or locally produced guidance does not assure that the same evidence-based practice is being implemented across the country.

Our study had several limitations. We opted for a pragmatic approach to this preliminary survey of HPTs as this was an easily accessible source of data, aiming to explore the potential burden of scabies cases and outbreaks associated with domiciliary care in England and understand how these outbreaks are managed from a public health perspective. Capturing data from free-text notes on the PHE case management system meant that data were sometimes missing and potential biases of the documenter could not be ascertained or avoided. Our approach also inevitably introduces selection bias as it is likely that only difficult outbreaks which community stakeholders are finding hard to manage will have been referred to HPTs for additional support, while smaller outbreaks or isolated episodes of transmission may not have been identified. However, the number and complexity of outbreaks that were captured in this study demonstrate that this is an issue of relevance to public health and community infection control and does warrant further investigation.

More research is required to better quantify the burden of scabies related to domiciliary care workers in the community and to understand the impacts it has on service users and the local health economy. This in turn will help inform good practice and help reduce any associated stigma. Infection control intervention studies are needed to provide evidence for best practice in the management of these outbreaks. More generally, further research is needed to evaluate the use of oral medicines to treat scabies as opposed to the currently recommended topical lotions which are particularly difficult to administer (particularly for the frail or confused) and unpleasant for the patient. Gaining licensure for systemic ivermectin treatments in the UK could make significant improvements to the accessibility and adherence to scabies treatment in the elderly. Better methods for diagnosing scabies, particularly in the elderly, are also required, and the development of a point of care test to replace current skin scraping and microscopy methods which lack sensitivity in this age group [5] would be ideal.

## Supplementary material

For supplementary material accompanying this paper visit https://doi.org/10.1017/S0950268819001274. click here to view supplementary material
