# Supplementary material for: The public health importance of scabies in community domiciliary care settings: an exploratory cross-sectional survey of Health Protection Teams in England
Source: Epidemiol Infect. 2019 Jul 10;147:e239. doi: 10.1017/S0950268819001274 (PMC6627010; doi:10.1017/S0950268819001274)
Supplement: Supplementary file 1 [file S0950268819001274sup001.docx]

**ONLINE SUPPLEMENT**

1. Survey invitation email

Dear colleagues,

Thames Valley Health Protection Team have been awarded funding from the NIHR Health Protection Research Unit in Emerging and Zoonotic Infections to undertake a research project in to the burden of scabies outbreaks affecting domiciliary care workers and service users in England.

The first arm of the study is a survey of health protection teams to quantify the burden of cases and outbreaks managed, and identify ways in which the management of scabies by domiciliary care agencies and other stakeholders can be improved. We need to recruit a volunteer from each health protection team in England to complete the survey to capture data on the number of cases of scabies related to domiciliary care that are notified to health protection teams, and the public health actions that were taken. It will require the volunteer to run a pre-specified query on HPZone and then fill in the survey tool; this should all take no longer than an hour.

This project supports the PHE objective of promoting research and development within the organisation, and will be the first research of its kind in to the unique context of scabies related to domiciliary care in England. It will increase our knowledge of scabies management and help us work towards more effective and efficient health protection actions.

We would be very grateful if you could identify a practitioner or registrar within your team to complete the survey and forward their email address to Emily Phipps (Emily.phipps@phe.gov.uk) who will liaise with them and provide the survey link. Could this please be completed by Tuesday 31st July.

Best wishes,

Emily

1. Survey items

This case definition and search strategy have been provided in the introductory email.

Cases have been included as well as situations as some may not have been converted to situations. Enquiries have not been included due to the volume of scabies enquiries handled by HPTs and the difficulty this presents in identifying those related to domiciliary care workers.

- CASE DEFINITION
- Any case or situation involving a confirmed, probable or possible case of scabies
- Notified between 1st January 2013 and 31st December 2017
- Where a case is a domiciliary care worker or receives care from domiciliary care services
- SEARCH STRATEGY
- Query type: case
- Infection: scabies
- Date entered: year beginning 2013
- Query type: situation
- Infectious agent: scabies mite
- Date entered: year beginning 2013
- HPZone number
- Date notified
- Date closed
- Number of events on case/situation
- Public health actions taken (tick all that apply)
  - Advice given regarding diagnosis
  - Advice given regarding treatment
  - Advice given regarding cleaning
  - Advice given regarding exclusion
  - Outbreak control team assembled
- Treatment provided (staff and service users)
  - Permethrin
  - Malathion
  - Permethrin & malathion
  - Ivermectin
  - Unknown
  - None
- Reported length of time between symptom onset and notification
- Reported number of care workers affected
- Reported number of service users affected
- Did you refer to any national or local guidelines? Please state name/author of guidance if known
- Who was liaised with and provided with guidance? If a multi-agency approach was adopted to manage the case/situation, please tick all stakeholders involved.
  - GP (of service user)
  - GP (of carer)
  - Care agency
  - Individual service user
  - Individual carer
  - CCG
  - Other (please state)
- Was co-ordinated treatment of multiple cases advised? (yes/no/unknown)
- Was co-ordinated treatment implemented? (yes/no/unknown)
- How was treatment of staff funded?
  - Self funded
  - CCG
  - Care agency
  - Unknown
  - Other (please state)
- What worked well?
- What didn’t work well?
- Any further comments?
